# Putative Inflammatory Sensitive Mechanisms Underlying Risk or Resilience to Social Stress

**DOI:** 10.3389/fnbeh.2018.00240

**Published:** 2018-10-26

**Authors:** Julie E. Finnell, Susan K. Wood

**Affiliations:** ^1^Department of Pharmacology, Physiology, and Neuroscience, University of South Carolina School of Medicine, Columbia, SC, United States; ^2^WJB Dorn Veterans Administration Medical Center, Columbia, SC, United States

**Keywords:** stress susceptibility, neuroinflammation, depression, microglia, glutamate

## Abstract

It has been well recognized that exposure to stress can lead to the onset of psychosocial disorders such as depression. While there are a number of antidepressant therapies currently available and despite producing immediate neurochemical alterations, they require weeks of continuous use in order to exhibit antidepressant efficacy. Moreover, up to 30% of patients do not respond to typical antidepressants, suggesting that our understanding of the pathophysiology underlying stress-induced depression is still limited. In recent years inflammation has become a major focus in the study of depression as several clinical and preclinical studies have demonstrated that peripheral and central inflammatory mediators, including interleukin (IL)-1β, are elevated in depressed patients. Moreover, it has been suggested that inflammation and particularly neuroinflammation may be a direct and immediate link in the emergence of stress-induced depression due to the broad neural and glial effects that are elicited by proinflammatory cytokines. Importantly, individual differences in inflammatory reactivity may further explain why certain individuals exhibit differing susceptibility to the consequences of stress. In this review article, we discuss sources of individual differences such as age, sex and coping mechanisms that are likely sources of distinct changes in stress-induced neuroimmune factors and highlight putative sources of exaggerated neuroinflammation in susceptible individuals. Furthermore, we review the current literature of specific neural and glial mechanisms that are regulated by stress and inflammation including mitochondrial function, oxidative stress and mechanisms of glutamate excitotoxicity. Taken together, the impetus for this review is to move towards a better understanding of mechanisms regulated by inflammatory cytokines and chemokines that are capable of contributing to the emergence of depressive-like behaviors in susceptible individuals.

## Introduction

Depression is considered to be one of the most debilitating diseases in the United States (Almeida, 2005) and has been globally recognized as a significant source of disability (Reddy, 2010). The prevalence of depression has been steadily increasing over the last 10 years from 6.6% to 7.3% in adults and 8.7%–12.7% in adolescents (Weinberger et al., 2017). While there are a number of available antidepressant therapies, many like the selective serotonin re-uptake inhibitor citalopram, are only 33% effective in producing full remission of depressive symptoms (Trivedi et al., 2006). Moreover, up to 30% of depressed patients are resistant to traditional antidepressant therapies (Joffe et al., 1996; Al-Harbi, 2012). These data strongly suggest that the pathophysiology underlying the emergence of depression is variable between individuals and is still largely unclear. It was first noted that activation of the immune system impacted psychiatric functioning back in 1927 when Julius Wagner-Jauregg won the Nobel Prize for this seminal observation. Since this initial discovery, there has been a striking increase in the number of publications on the topic of inflammation related depression (Loftis et al., 2010). These studies have demonstrated that certain subpopulations of depressed patients exhibit greater levels of interleukin (IL)-6 and C reactive protein (CRP) in the plasma (Irwin and Miller, 2007) and cerebrospinal fluid (Sasayama et al., 2013; Devorak et al., 2015). Importantly, this vast body of literature has also established a causal link between inflammation and depression. Several clinical studies demonstrated that chronic administration of the cytokines interferon (INF)-α and IL-2 as chemotherapeutics were capable of inducing depression in a large number of patients (Denicoff et al., 1987; Renault et al., 1987). Moreover, it should be noted that individuals with inflammatory diseases such as irritable bowel disease, allergic rhinitis and rheumatoid arthritis (Cuffel et al., 1999; Stauder and Kovács, 2003; Katon et al., 2004; Marrie et al., 2017) as well as cardiovascular disease (Anda et al., 1993; Riba et al., 2011; Huffman et al., 2013) are at increased risk of developing psychiatric comorbidities.

Beyond immune diseases as a risk factor for psychiatric disorders, it has been well established that exposure to stress can also serve as an independent risk factor for the emergence of psychosocial disorders. While there are many different types of stress, social stressors such as bullying, abuse, isolation, witnessing traumatic events, or taking care of a terminally ill loved one are the most common types of stress encountered by people (Almeida, 2005). Importantly, it has been shown that exposure to social stress can not only produce increases in markers of inflammatory activity (Slavich et al., 2010; Allen et al., 2017) but can also augment underlying inflammatory disorders including allergic responses (Sandberg et al., 2000; Liu et al., 2002; Kiecolt-Glaser et al., 2008). However, preclinical and clinical studies have shown that there is considerable individual variability in the behavioral and inflammatory consequences induced by stress exposure resulting in the emergence of resilient and susceptible subpopulations. Specifically, it has been shown that greater inflammatory responses to stress are associated with greater negative affect in humans (Dickerson et al., 2009) and promote the development of depressive-like behaviors in rodents (Wohleb et al., 2013, 2014a,b; Hodes et al., 2014; Wood et al., 2015; Finnell and Wood, 2016; Finnell et al., 2017a,b, 2018). These stress-induced inflammatory effects are known to extend well beyond the immediate response to stress such that late phase inflammatory responses are also enhanced following social stress exposure (Kiecolt-Glaser et al., 2008; Deak et al., 2017). These late phase inflammatory effects have been tied to the emergence of chronic elevations of inflammatory factors through the recruitment and sensitization of inflammatory competent cell types including peripherally derived T cells (Janeway et al., 2001; Hansen et al., 2004) and microglia (Badoer, 2010).

Activation or sensitization of microglia, the resident immune cells of the brain, is of particular relevance to depression as a recent clinical study showed for the first time that depressed patients exhibit significant increases in translocator protein density, a marker of activated microglia (Setiawan et al., 2015). Under normal resting conditions, microglia exhibit a highly ramified morphology that is associated with monitoring and maintenance of the neural cell microenvironment (Nimmerjahn et al., 2005; Kettenmann et al., 2011). In response to a stress or immune challenge, these cell types take on an ameboid morphology that is associated with a reactive inflammatory state (Gemma and Bachstetter, 2013; Brites and Fernandes, 2015) resulting in the release of a number of different effectors including cytokines and chemokines (Brites and Fernandes, 2015). In this way reactive microglia are known to propagate inflammatory signals throughout the brain (Fruhbeis et al., 2013). However, the discrete neural mechanisms that may be impacted by the release of cytokines and chemokines in susceptible individuals remains unclear. Therefore, the focus of this review is to first provide an overview of the sources of individual differences in stress and inflammatory responses and second, to highlight discrete neural and glial mechanisms that are regulated by inflammatory effectors that may contribute to the emergence of behavioral dysfunction associated with a depressive-like state. Great focus has been placed on clinical and preclinical studies documenting the effects of social stress. However, other modalities of stress are discussed in instances where literature using social stress models is lacking.

## Sources of Individual Differences in Inflammatory Stress Responses

Prior to beginning a discussion on the discrete neural mechanisms that may underlie the emergence of inflammatory related depressive-like behavior, it is critical to understand how individual factors such as age, sex and inherent differences in personality or coping may differentially impact the inflammatory system thereby contributing to stress susceptibility or resiliency.

### Age

Stress susceptibility is well known to change across the lifespan. Importantly, life stages in which the brain is undergoing significant alterations, such as neural development and maturation in the young and senescence in the elderly (Graham et al., 2006), are associated with heightened susceptibility to the consequences of stress exposure. Much like stress susceptibility, immune function is also known to change across the lifespan. In general, innate and adaptive immune function decreases as individuals age (Lord et al., 2001; Gomez et al., 2005), resulting in dysregulated inflammatory responses to stress or immune challenges (Lord et al., 2001). For example, studies in rodents have indicated that aged rats do not develop inflammatory tolerance to repeated lipopolysaccharide (LPS) injections as is observed in younger rats (Li et al., 2009). Moreover, LPS inflammatory reactivity has also been shown to be greater in middle-aged mice compared with young mice (Kohman et al., 2010). This increase in inflammatory reactivity in aged animals has also been demonstrated in the brain as a result of natural microglial shifts towards a “primed” phenotype (Barrientos et al., 2015). Heightened inflammatory sensitivity in aging populations, termed inflammatory senescence, also extends to the inflammatory response to stress. Specifically, it has been shown that transient stressors more commonly produce maladaptive inflammatory responses in the elderly compared to younger individuals (Segerstrom and Miller, 2004). Moreover, exposure to stress can also accelerate the process of inflammatory senescence. This assumption is supported by a prospective clinical study which determined that older adults serving as care givers exhibited a four-fold faster elevation in resting plasma IL-6 over a 6-year period compared to age-matched non-caregivers (Kiecolt-Glaser et al., 2003). While clinical studies assessing stress responsivity in aging populations are relatively limited, it is well recognized that social stress and particularly social isolation is extremely common especially for those living in retirement communities. This is of particular importance as approximately 15% of elderly individuals living in retirement communities exhibit significant depressive symptomatology and are more likely to exhibit suicidal tendencies (Fiske et al., 2009). Based on the strong role that stress-induced inflammation is suggested to play in the emergence of depressive-like behavioral states, it is possible that inflammatory senescence may represent a putative mechanism underlying the emergence of depression in aged populations.

Younger populations on the other hand generally exhibit greater resilience to immune challenges while simultaneously exhibiting enhanced behavioral susceptibility to stress. At a cursory glance these effects seem to be opposing. However, these data do not consider the detrimental effects that inflammation produces in the developing organism. Specifically, it has been shown that stress (Bath et al., 2016) and inflammation (Johnson and Kaffman, 2018) at early developmental stages can significantly alter the function, maturation and proliferation of neurons and glia. Moreover, exposure to early life stress is known to promote shifts in the function of immune cells that are resistant to alterations later in life (Lubach et al., 1995), suggesting that early life stress results in long-term reprogramming of the immune system. This assumption has been verified by several studies demonstrating that early life stress not only increases the susceptibility to developing autoimmune deficiencies (Capitanio and Lerche, 1991) but also produces sensitization to subsequent immune challenges (Graham et al., 2006; Roque et al., 2014). Importantly, these shifts in immune function are known to persist for several years (Graham et al., 2006) and has the potential to persist into adulthood (Harry and Kraft, 2012; Delpech et al., 2016). This long-term reprogramming of the immune system has been suggested to underlie the emergence of depressive episodes in younger populations as subsequent stress exposures can produce augmented and poorly regulated physiological responses (Brown et al., 1977).

### Sex

Over the last two decades special attention has been paid to understanding the putative contribution of sex, and more specifically gonadal hormones, to the consequences of stress exposure. This research interest was facilitated by several clinical reports that documented that women are more likely to be diagnosed with depression compared with men (Weissman and Klerman, 1992; Gallo et al., 1993; Kessler et al., 1993; Hankin et al., 1998). This two-fold increased risk is known to emerge at the onset of puberty, persists into adulthood (Kessler et al., 1993; Hankin et al., 1998; Nolen-Hoeksema, 2001), and ends following menopause (Kessler et al., 1993; Hankin et al., 1998) strongly suggesting that ovarian hormones may mediate this enhanced stress susceptibility in females. It is important to note that under non-stress conditions, ovarian hormones have consistently been suggested to confer protection because ovariectomy increases depressive-like behaviors (Li et al., 2014). However, when gonadally-intact and ovariectomized female mice are exposed to repeated stress, ovariectomy confers protection against stress-induced depressive-like behavior (LaPlant et al., 2009). Ovarian hormones, like androgens in males, exert control over a number of physiological systems including inflammation (Villa et al., 2016). This is of particular importance as women exhibit greater inflammatory-induced depressive behaviors following an acute endotoxin challenge compared to men (Moieni et al., 2015). Importantly, this ovarian hormone mediated control over inflammatory systems has also been reported in preclinical models demonstrating that female mice exhibit a greater number of microglia that also exhibit more reactive morphology in brain areas associated with emotional regulation (Schwarz et al., 2012). Moreover, when estrogen is administered *in vivo* and microglia are subsequently cultured, microglia with prior estrogen treatment are sensitized to LPS stimulation (Loram et al., 2012). However, it should be noted that the effect of estrogen on microglia have also been demonstrated to suppress cytokine release, but only when estrogen is applied *ex vivo* to microglial cells in culture (Dimayuga et al., 2005; Loram et al., 2012).

One of the most common forms of social stress conducted in the laboratory setting is the resident intruder paradigm of social defeat originally developed by Miczek (1979). Social defeat capitalizes on the protection and defense of territory. This model of social stress has proven to be very effective in males and readily produces anxiety- and depressive-like behaviors in the intruder rats (Wood et al., 2010, 2013, 2015; Chaijale et al., 2013; Patki et al., 2013; Finnell et al., 2017a). However, running social defeat in female rats can be difficult and requires either a lactating female resident (Jacobson-Pick et al., 2013) or modification of the male resident with DREADDs to induce heightened aggression via activation of the ventromedial hypothalamus (Takahashi et al., 2017). Recently a new modification to the resident intruder paradigm has also been conducted in which aggression by the male resident was induced following the application of male odorants to the female intruders (Harris et al., 2018). Exposure to this particular modality of social stress (i.e., defeat by a male resident) in female rats has produced incongruent results (Haller et al., 1999; Huhman et al., 2003; Shimamoto et al., 2011; Trainor et al., 2011; Holly et al., 2012; Greenberg et al., 2013, 2015; Jacobson-Pick et al., 2013; Ver Hoeve et al., 2013; Takahashi et al., 2017; Harris et al., 2018). In contrast, findings from the Trainor lab have consistently demonstrated that female California mice display greater sensitivity to the behavioral and molecular consequences to social defeat stress compared with males (Trainor et al., 2011; Greenberg et al., 2013, 2015; Duque-Wilckens et al., 2018). These species dependent effects of social defeat stress in females may underscore the ethological relevance of this stress modality. Unlike female rats which demonstrate territorial aggression only during the lactation period, female California mice inherently demonstrate territorial aggression. These data suggest that the physical interaction of social defeat may be more ethologically relevant in female/male California mice and male rats compared with female rats. This assumption is further validated by studies demonstrating that female rats exhibit greater sensitivity to social isolation/instability compared with social defeat (Haller et al., 1999).

With this in mind, a new model of social stress has recently emerged that combines the olfactory, auditory and visual exposure of social defeat without requiring the physical interaction of defeat. Using this vicarious witness stress model originally developed for use in male mice by Warren et al. (2013), we have shown that intact female rats demonstrate greater sensitivity to the inflammatory, cardiovascular and behavioral consequences of witness stress exposure compared to ovariectomized female rats (Finnell et al., 2018). We have further demonstrated that this enhanced and prolonged behavioral and physiological sensitivity to the consequences of witness stress is not exhibited to the same extent in male rats (Finnell et al., 2017b). While this is still a relatively new model of stress, others have also been able to demonstrate similar behavioral sensitivity of intact female mice to this vicarious witness stress exposure (Iniguez et al., 2018), suggesting that female susceptibility to witness stress may be conserved across species. In humans, bearing witness to a major stressor is one type of event that can elicit post traumatic stress disorder (PTSD). Therefore, it should be noted that similar to findings in depressed patients, PTSD in the clinical setting is also associated with a significant shift in immune reactivity (reviewed in Segerstrom and Miller, 2004). Interestingly, this immune reactivity differs between men and women with men exhibiting a general under-expression of inflammatory related genes of collected CD14^+^ monocytes while women exhibit an upregulation of pathways associated with inflammatory activation (Neylan et al., 2011). Several comprehensive reviews have recently been published regarding enhanced stress sensitivity and increased risk of mood disorders in females (Goel and Bale, 2009; Bangasser and Wicks, 2017; Bangasser and Wiersielis, 2018; Wickens et al., 2018). Moving forward it will be critical to further validate whether stress sensitive mechanisms in females are mediated in part by inflammatory processes.

### Personality and Coping

It has long been recognized that there is wide variability in the way people process and assess stressful situations (Lupien et al., 2007). This may be driven by the individual’s cognitive interpretation (Lupien et al., 2007; Nicolai et al., 2013) as well as the behavioral coping mechanism that is adopted during the stress exposure. In general, coping strategies are broadly classified into two categories termed passive and active. Passive coping strategies include avoidance, seeking excessive reassurance, withdrawal and substance abuse (Cambron et al., 2009; Cairns et al., 2014). In contrast, active coping strategies include problem solving, seeking support, exercising and engaging in adaptive processes (Cairns et al., 2014). It is understood that the coping response adopted by an individual will vary depending on the type and severity of the stress exposure. However, it has been suggested that individuals who more readily adopt active coping strategies are more likely to be resilient to the behavioral and physiological consequences of stress compared to those who more readily adopt passive coping strategies (Kendler et al., 1991). Importantly, coping responses have also been shown to play a large role in the inflammatory outcomes of stress. For example, individuals who more readily adopt passive coping strategies exhibit greater plasma concentrations of IL-6 following a 3 min simulated public speaking challenge compared with individuals that adopt active coping strategies (Carroll et al., 2011). Additionally, feelings of helplessness during stress exposure are associated with sensitized immune responses to a common allergen and promote greater release of IL-6 from stimulated primary blood leucocytes (Kiecolt-Glaser et al., 2009). These data suggest that feelings of helplessness or uncontrollability could promote sensitization of inflammatory pathways that can be amplified by stress exposure (Chen et al., 2009). Although it is impossible to truly assess the emotional state of an animal, a number of preclinical studies demonstrated that both coping (Koolhaas et al., 1999, 2007; Sih et al., 2004; Bell, 2007; Wood et al., 2015; Finnell and Wood, 2016) and stressor controllability (Gray and Cooney, 1982; Frank et al., 2007; Christianson et al., 2009; Arakawa et al., 2014) are large factors in the susceptibility for developing stress-induced behavioral and inflammatory dysfunction. Several recent reviews have also been published on the topic of stress coping and inflammatory outcomes (Maier and Watkins, 2005; Koolhaas et al., 2007; Wood, 2014; Finnell and Wood, 2016; Wood et al., 2017).

## Brain Areas Associated With Stress Susceptibility and Resiliency

There are a number of brain regions that have been implicated in the emergence of stress-induced behavioral dysfunction that are discussed throughout this review. Several extensive reviews have been published on this topic, for example (McEwen and Gianaros, 2010). However, to highlight the importance of the brain regions described herein, we have included a brief overview of the brain areas that are associated with social processing and stress responses.

### Prefrontal Cortex

The prefrontal cortex works to integrate the social, emotional and cognitive aspects of behavior (Satpute and Lieberman, 2006). Dysfunction within the prefrontal cortex in humans has been associated with the emergence of socially inappropriate behaviors, apathy, inflexibility and isolation (Barrash et al., 2000). In addition to producing overall shifts in social behavior, the prefrontal cortex has also been implicated in stress-induced coping strategies (Robinson et al., 2015). Similar associations between prefrontal cortex activation and susceptibility to the consequences of stress have also been demonstrated in rodents. Utilizing chronic social defeat stress in mice, it was shown that individual susceptibility to the behavioral effects of chronic social defeat (i.e., social avoidance) was directly associated with the activity of the prefrontal cortex (Kumar et al., 2014). Moreover, Kumar et al. (2014) went on to demonstrate that prefrontal cortex reactivity during a pre-stress forced interaction test was predictive of individual stress susceptibility following chronic social defeat. In the context of emotional regulation and threat assessment, the prefrontal cortex serves as a top down inhibitory regulator of the amygdala and hypothalamus (Mujica-Parodi et al., 2017). Several clinical and preclinical studies have consistently reported that stress-induced behavioral deficits are often associated with dendritic atrophy, loss of synapses, and altered prefrontal cortex connectivity (Radley and Morrison, 2005; Banasr et al., 2007; Drevets et al., 2008; Radley et al., 2008; Ota et al., 2014). These morphological and physiological alterations of prefrontal cortex neurons may therefore result in disinhibition of downstream signaling targets including the amygdala.

### Hippocampus

Although largely known for its role in declarative memory, the hippocampus has also been implicated in social and emotional episodic memories (Dolcos et al., 2017). Through its connectivity and bottom-up signaling with the amygdala, the hippocampus is responsible for the encoding and retrieval of emotionally laden memories (Dolcos et al., 2017). In addition, the hippocampus is also critical for the re-encoding and extinction of these memories. Exposure to chronic unpredictable restraint stress was shown to produce reductions in several hippocampal sub regions including CA1, CA3 and the dentage gyrus (Schoenfeld et al., 2017). Reductions of hippocampal volume in response to stress have been associated with both dendritic atrophy (Watanabe et al., 1992; Wood et al., 2004; Eiland and McEwen, 2012) and reduced neurogenesis (Simon et al., 2005; Jayatissa et al., 2006; Mitra et al., 2006; Schoenfeld et al., 2017). Interestingly, preclinical studies using social defeat in mice have indicated that defeat-induced reductions of neurogenesis within the hippocampus is associated with stress susceptibility (Tse et al., 2014) and mice demonstrating stress resiliency exhibited an increase of hippocampal neurogenesis by approximately 4% (Tse et al., 2014).

### Amygdala

The amygdala is best known for its role in fear responses. For example animals with lesions of the amygdala exhibit a disinhibition of fear responses and a significant increase in prosocial behavior (Kluver and Bucy, 1997). Chronic stress is also known to produce significant structural and functional effects within the amygdala that are highly dependent upon the type and duration of the stressor (Wilson et al., 2015). Moreover, it is now well recognized that depression and anxiety are both associated with amygdala hyperactivity (Drevets, 2000; Sheline et al., 2001). While it is currently unclear how active and passive stress coping strategies are associated with amygdala activity, it has been shown using rodent models that resilient individuals exhibit a number of stress-induced adaptations that may inhibit over activation of the amygdala (Silveira Villarroel et al., 2018).

### Bed Nucleus of the Stria Terminalis

Considered to be a part of the extended amygdala, the bed nucleus of the stria terminalis (BNST) is best known for its involvement in behaviors associated with social bonding (Coria-Avila et al., 2014), aggression (Nelson and Trainor, 2007), mating (Coria-Avila et al., 2014) and stress-induced cardiovascular function (Crestani et al., 2013; Oliveira et al., 2015). Interestingly, fMRI studies in humans have determined that the BNST is also involved in the generation of anticipatory anxiety to unpredictable noxious stimuli (Straube et al., 2007; Alvarez et al., 2011; Yassa et al., 2012). Although it is currently unclear how stress affects BNST function in humans, studies using rodent models have determined that exposure to stress results in enhanced BNST activation (Kollack-Walker et al., 1997; Martinez et al., 1998). It is also important to note that the BNST is a sexually dimorphic brain region that has been shown to play a critical role in the consequences of social defeat exposure in male and female monogamous California mice. Following social defeat exposure, Greenberg et al. (2013) showed that female California mice not only demonstrated greater social avoidance but also exhibited greater brain derived neurotrophic factor in the BNST compared to their defeated male counterparts.

### Nucleus Accumbens

The Nucleus Accumbens (NAc) is largely studied in the field of addiction due to its role in motivation and reward. However, the NAc is quickly gaining attention in the field of stress and depression due to its potential involvement in the development of anhedonia (Di Chiara et al., 1999; Yadid et al., 2001). The NAc is predominantly inhibitory, releasing γ-aminobutylic acid (GABA) in the ventral tegmental area, thereby exerting control over cortical dopamine (Shirayama and Chaki, 2006). fMRI studies conducted in humans have shown that patients suffering from major depressive disorder exhibit altered activation of the NAc during reward anticipation and outcome compared to healthy controls (Misaki et al., 2016). Importantly, the NAc has also been implicated in coping behaviors. Use of active coping behaviors was associated with increases in NAc activity while passive coping was association with reductions in NAc activity (Levita et al., 2012).

## Sources of Stress-Induced Neuroinflammation

**Figure 1:**
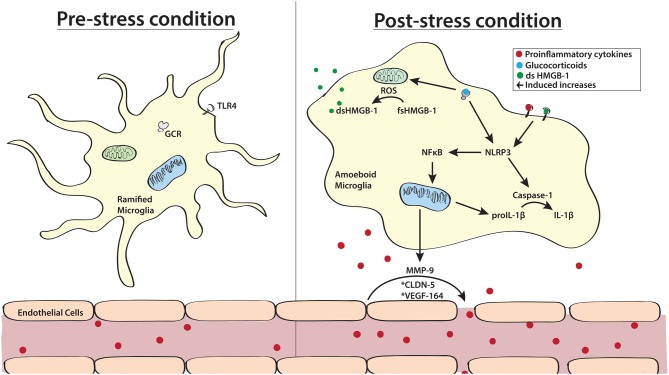
Schematic highlighting key sources of stress-induced neuroinflammation. Stress exposure is known to promote shifts in microglial morphology from a highly ramified “resting” state to an ameboid M1 proinflammatory state. In addition to directly stimulating the release of cytokines, activation of microglial glucocorticoid receptors (GCRs) also results in priming of inflammatory responses. This process can occur directly through activation of the NLRP3 inflammasome or indirectly by promoting the release of reactive oxygen species (ROS) from mitochondria which results in the oxidation of high mobility group box -1 (HMGB-l). Once released, HMGB-1 and proinflammatory cytokines such as interleukin (IL)-1β can act on toll like receptor 4 (TLR 4) on the surface of microglia to further stimulate the NLRP3 signaling cascade. Another significant source of stress-induced neuroinflammation is the breakdown of the blood brain barrier (BBB). In pre-stress conditions endothelial cells tightly adhere to one another, blocking the flow of circulating cytokines to the brain. However, in response to stress exposure, tight junctions between these endothelial cells break down allowing for peripheral cytokines and inflammatory cells to penetrate into the brain. This process is known to be facilitated by plasma vascular endothelial growth factor (VEFG)-164, endothelial claudin-5 (CLDN-5) and microglia released matrix metalloproteinase-9 (MMP-9). *Designate non-neuronal and non-glial origins.

While it is clear that neuroinflammatory processes may be a critical link in the pathogenesis of stress-related psychiatric disorders in certain subpopulations of patients, it is important to understand which stress sensitive processes are capable of promoting neuroinflammation. The two most likely mechanisms of increased neuroinflammation include stress-induced activation and sensitization (i.e., priming) of microglia and stress-induced disruption of the blood brain barrier (BBB; see Figure 1).

### Microglial Activation and Priming

Microglia are considered to be highly adaptive cell types as they are capable of transitioning between pro-inflammatory (M1) and anti-inflammatory (M2) states. However, in response to stress a greater number of microglia exhibit the proinflammatory M1 phenotype (Tang et al., 2018). This change in morphology can be stimulated by activation of glucocorticoid receptors (GCRs; Ros-Bernal et al., 2011; Liu et al., 2016) found on the cell surface of microglia, suggesting that stress-induced release of cortisol (in humans) and corticosterone (in rodents) could promote this shift to an M1 microglial state. The involvement of M1 type microglia in stress-induced neuroinflammation has further been supported by studies utilizing the tetracycline analog minocycline. Minocycline, traditionally used as an antibiotic, is well documented to inhibit the polarization of microglia to an M1 proinflammatory phenotype (Kobayashi et al., 2013). Moreover, use of minocycline in conjunction with inescapable foot shock (Blandino et al., 2006) and chronic mild stress (Wang et al., 2018) have shown that inhibition of the M1 microglial phenotype, and subsequent suppression of proinflammatory cytokine release, protects against the development of stress-induced depressive- and anxiety-like responses in rodents. Notably, minocycline is now being evaluated as a putative treatment for bipolar depressive disorder in humans. A very recent clinical trial demonstrated that daily doses of minocycline was capable of producing anti-depressant effects in 90% of study participants (Murrough et al., 2018). While more information is required about the putative anti-inflammatory effects minocycline may have in these patients, these studies in combination provide clear evidence for the involvement of M1 microglia in the emergence of depressive symptomatology.

In addition to promoting the release of cytokines from microglia (Nair and Bonneau, 2006; Kreisel et al., 2014), stress is also capable of sensitizing microglia such that a subsequent stress or immune challenge produces a faster and more robust neuroinflammatory response (Frank et al., 2012, 2018; Fonken et al., 2016). Importantly, glucocorticoid signaling is one factor that has been shown to initiate this phase of stress-induced neuroinflammatory sensitization termed microglial priming. One potential mechanism by which this priming may occur is though the dysregulation of the danger, damage and disease signal high mobility group box-1 (HMGB-1). In response to stress, the membrane glycoprotein CD200 and its receptor (CD200R) exhibit significant down regulation at both the genomic and protein levels (Frank et al., 2018). Notably CD200R is almost exclusively expressed on microglia (Koning et al., 2009) and is known to regulate proinflammatory signaling by constitutively inhibiting myloid cells (Gorczynski, 2005). Loss of CD200 and CD200R in rats exposed to inescapable foot shock was further associated with enhancement of HMGB-1 and increased gene expression of IL-1β, tumor necrosis factor (TNF)-α and nuclear factor kappa (NFκ)B (Frank et al., 2018). This increased expression of proinflammatory genes by HMGB-1 has been directly linked to the activation of the nucleotide-binding oligomerization domain-like receptor (NLRP3) inflammasome (Weber et al., 2015). In addition to the noted effects on gene expression, HMGB-1 activation of the NLRP3 inflammasome can further potentiate proinflammatory signaling by enhancing the cleavage of proIL-1β to IL-1β via activation of caspase-1 (Yan et al., 2012). However, it is important to note that this proinflammatory capacity of HMGB-1 is strongly tied to the redox state of the protein. In its fully reduced state, HMGB-1 promotes chemotaxis but lacks the ability to promote proinflammatory signaling. Alternatively, the oxidized state of HMGB-1, designated by the formation of disulfide linkages, is capable of potentiating proinflammatory signaling as discussed above but lacks chemotactic abilities (Yang et al., 2012). Although the majority of studies assessing the involvement of HMGB-1 in microglial priming have come from studies using inescapable foot shock (Yang et al., 2012; Weber et al., 2015), chronic unpredictable stress (Franklin et al., 2018), and single prolonged stress (Lai et al., 2018), exposure to social stressors such as social defeat is known to enhance the intracellular concentration of reactive oxygen species (ROS; see section Oxidative Stress/Reactive Oxygen Species). Therefore, it is highly plausible that HMGB-1 may also contribute to the emergence of social stress-induced behavioral deficits.

These stress-induced alterations in the morphology and reactivity of microglia requires several hours to manifest and are evident for up to 72 h following the termination of stress, a time point at which peripheral cytokine responses are no longer detected (Tynan et al., 2010; Kopp et al., 2013; Deak et al., 2017). These data nicely parallel findings indicating that the development of depressive-like behaviors following a robust inflammatory challenge occurs over a period of several hours and persist well beyond 24 h (Capuron et al., 2002; Dantzer et al., 2008). Moreover, preclinical studies using social defeat and vicarious witness stress have demonstrated that repeated stress exposure is capable of enhancing resting neuroinflammation that persists for at least 5 days following the final stress exposure, a time at which depressive-like behavior is evident (Finnell et al., 2017a,b). Importantly these studies determined that despite elevations in neuroinflammation and depressive-like behavior 5 days following the final stress exposure, resting peripheral inflammation had returned to baseline comparable to non-stressed controls (Finnell et al., 2017a). The importance of central inflammation in the emergence of stress-induced depressive-like behavior has been further substantiated by studies outlining the effectiveness of centrally administered IL-1 receptor antagonist in inhibiting social defeat-induced depressive-like behavior (Wood et al., 2015). Similar antidepressant-like effects were demonstrated with the use of resveratrol, a natural anti-inflammatory. Importantly, these effects were only achieved by the highest dose, which was the only dose to effectively prevent the neuroinflammatory response to social defeat (Finnell et al., 2017a). These data strongly suggest that stress likely promotes the emergence of an M1 microglial phenotype which may directly underlie stress and inflammatory-induced behavioral dysfunction.

### Blood Brain Barrier Disruption

While cells within the brain are robustly capable of producing a major source of neuroinflammation, cytokines circulating in the blood can also serve as a source to increase neuroinflammation. The BBB, in part a meshwork of specialized endothelial cells along blood vessels surrounding the brain, serves the purpose of regulating entry and export of cytokines (and other substances) between the peripheral circulation and the brain. In a healthy brain, cytokines are considered to be too large and hydrophilic to passively diffuse across the BBB (Banks, 2005). However, the IL-1 family, TNF and IL-6 exhibit distinct and saturable transport mechanisms to effectively pass from the blood to the brain (Banks et al., 1989, 1991). Moreover, pro-inflammatory cytokines can disrupt the integrity of the BBB (Muramatsu et al., 2012). As such, circulating inflammation may initiate a cascade that enhances the flow of inflammatory factors from the circulation into the brain, further exacerbating neuroinflammation. This concept was demonstrated in an elegant study that showed that microglia initiated the recruitment of IL-1β producing monocytes to the brain and stimulated brain endothelial IL-1R1 (McKim et al., 2018). This study went on to further demonstrate that microglial depletion prevented monocyte recruitment and inhibited the development of anxiety in socially defeated mice.

It is tempting to suggest that the link between diseases characterized by peripheral inflammation including cardiovascular disease, rheumatoid arthritis, etc., and the striking increased risk of major depression in these patients (Anda et al., 1993; Huffman et al., 2013; Marrie et al., 2017) may be driven by an impaired BBB and exaggerated neuroinflammation. In addition, social stress exposure, another risk factor for psychiatric disorders is recognized to increase the release of circulating proinflammatory cytokines in animals and humans (Pace et al., 2006; Hodes et al., 2014; Wood et al., 2015; Quinn et al., 2018). While circulating cytokine levels typically return to baseline following cessation of a single acute social stressor (Cheng et al., 2015), preclinical models generating a stress-induced depressive-like phenotype achieved by repeated exposure to social defeat stress demonstrate persistent enhancement in peripheral inflammatory sensitivity (Hodes et al., 2014; Finnell et al., 2017a). In line with the deleterious role of pro-inflammatory cytokines on the integrity of the BBB, recent reports have identified the role of social stress on various factors known to disrupt the BBB. For example, male rats that demonstrate susceptibility to social defeat stress as evidenced by passive coping responses during social defeat and development of depressive-like behaviors, selectively demonstrated enhanced BBB permeability in the ventral hippocampus (Pearson-Leary et al., 2017) while the active coping resilient subset of rats did not. Moreover, administration of the proinflammatory cytokine vascular endothelial growth factor-164 increased permeability of the BBB and was shown to induce vulnerability in socially defeat rats (Pearson-Leary et al., 2017). Stress-induced BBB disruption has also been documented in a mouse model of social defeat, whereby the susceptible subset of male mice also demonstrated stress-induced suppression of claudin-5, an endothelial cell-specific tight junction protein, in the NAc and the hippocampus as compared with controls or the resilient subset of mice. Moreover, BBB permeability was also confirmed in the susceptible subset of mice (Menard et al., 2017). Importantly, these studies further established suppressed claudin-5 expression in post mortem tissue from the NAc of depressed patients. Taken together, disruption of the BBB is a likely susceptibility mechanism driving increased neuroinflammation and social stress-induced behavioral dysfunction in animals, and may contribute to psychopathology in humans.

Other proteins are likely targets for stress-induced increases in BBB permeability and include HMGB-1 and matrix metalloproteinase-9 (MMP-9). For example, HMGB-1 is upregulated by social defeat stress (Finnell et al., 2017b) and beyond its role in neuroinflammatory priming, is also involved in BBB dysfunction. This role for HMGB-1 is supported by studies demonstrating that administration of monoclonal antibody to HMGB-1 protects against ischemia-induced BBB disruption in rats (Zhang et al., 2011), and in humans anti-HMGB1 monoclonal antibody improves the BBB integrity of patients with Alzheimers disease (Festoff et al., 2016). Together these findings clearly define a role for HMGB-1 in BBB dysfunction that could precipitate stress-related psychiatric dysfunction. Furthermore, inflammatory factors including HMGB-1 (Qiu et al., 2010) also stimulate the release of MMP-9, a zymogen that breaks down the BBB, from infiltrating leukocytes and microglia to contribute to endothelial damage (Crocker et al., 2006) and BBB leakage (Seo et al., 2013). MMP-9 protein expression is elevated in peripheral tissues and serum by social defeat stress (Stelzhammer et al., 2015; Wu et al., 2015). While this has yet to be documented in the brain following social defeat, central MMP-9 has been shown to be regulated by fear learning (Ganguly et al., 2013) and lends support to the possibility that MMP-9 may be a putative target by which social stress could lead to BBB disruption.

## Central Mechanisms Conferring Risk or Resilience to Stress That Are Regulated by Neuroinflammation

**Figure 2:**
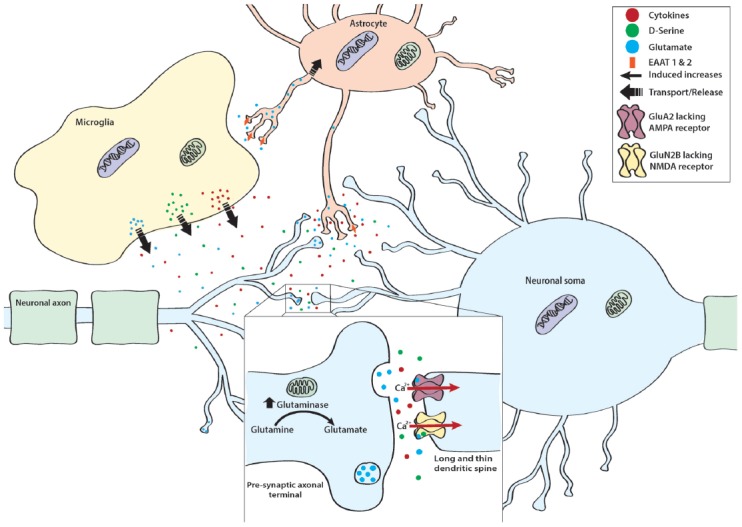
Mechanisms of stress-induced cytotoxicity. In addition to releasing cytokines, Ml type proinflammatory microglia release a variety of neurotransmitters, co-agonists, and neuromodulators such as glutamate and its co-agonist D-serine. Normally, excess glutamate is taken up by excitatory amino acid transporter (EAAT) 1 and 2 found on astrocytic processes. However, in proinflammatory conditions and in the presence of excess glutamate, EAAT 1 and 2 are down regulated, thereby resulting in excess glutamate within the synaptic cleft. Importantly, neurons also contribute to stress-induced enhancements of glutamatergic tone. This is thought to occur as stress exposure enhances mitochondrial glutaminase, the enzyme responsible for converting glutamine to glutamate. In addition to enhancing excitatory tone, stress also sensitizes neurons to the excitatory effect of glutamate. Specifically, stress promotes the expression of GluA2 lacking α-amino-3-hydroxy-5-methyl-4-isoxazolepropionic acid (AMPA) receptors and GLUN2B lacking NMDA receptors. These receptor subtypes allow for calcium (Ca^2+^) to freely pass into the cell thereby enhancing the depolarizing effect of glutamate. This cumulative increase in excitatory tone is particularly detrimental for dendritic spines that exhibit a long and thin morphology, as these spines are more sensitive to the degenerative effects induced by glutamatergic excitotoxicity.

Acute stress is well recognized to stimulate the release of proinflammatory cytokines from microglia (Blandino et al., 2006, 2013) and repeated stress exposure is capable of producing enduring increases in neuroinflammation in stress sensitive brain regions (Voorhees et al., 2013; Wohleb et al., 2013; Wood et al., 2015; Finnell et al., 2017a,b). While evidence links an inflammatory state with a depressive phenotype, our understanding of exactly which neuromodulatory systems are acted upon by inflammatory cytokines and chemokines that serve to increase stress susceptibility is in its infancy. Several reviews have been published on the impact that neuroinflammation has on the metabolism of the neurotransmitters serotonin, dopamine and glutamate and therefore, while clearly relevant to the pathophysiology of depression, this topic will not be covered here (see Miller et al., 2013). Herein, we will focus on the potential role of neuroinflammation on mitochondrial dysfunction and oxidative stress as well as glutamate neurotransmission or excitotoxicity (see Figure 2).

### Mitochondrial Dysfunction

Mitochondria play a critical role in cellular energy metabolism and supply the large energy demand required by the brain, especially under stressful conditions. The inner membrane of mitochondria houses the electron transport chain, which is made up of five protein complexes. Three of these respiratory chain complexes (I, III and IV) pump protons throughout the inner membrane generating the proton gradient that is ultimately responsible for synthesizing adenosine triphosphate (ATP) at complex V. Mitochondria are responsible for producing the vast majority of the ATP in neurons and in particular within presynaptic terminals mitochondrial ATP is required for synaptic ion homeostasis and phosphorylation (Mattson et al., 2008). There is mounting evidence that patients with psychiatric disorders demonstrate mitochondrial abnormalities at the functional level. For example, positron emission tomography studies of brain glucose metabolism have identified reduced glucose utilization in the brains of depressed patients (Videbech, 2000). Moreover, mitochondrial ATP production was also reduced in depressed patients (Gardner et al., 2003). While the cause of this mitochondrial dysfunction is not understood, it is noteworthy to consider the findings that proinflammatory cytokines can impair mitochondrial function. For example, physiologically relevant levels of TNF-α can induce mitochondrial dysfunction; low (post-stroke) levels of TNF-α rapidly reduce mitochondrial function as indicated by increased caspase 8 activity and a decrease in mitochondrial membrane potential (Doll et al., 2015). This effect was shown to signal through TNF-R1 selectively and highlights the role that proinflammatory cytokines may play in mitochondrial dysfunction. Beyond the capability of neuroinflammation to induce mitochondrial dysfunction, it is interesting to note that reducing activity of mitochondria within microglia amplifies the NLRP3 inflammasome and IL-1β release (Sarkar et al., 2017). Taken together these studies demonstrate a striking relationship between neuroinflammation and mitochondrial dysfunction.

While no studies to date have directly evaluated the role that stress-induced release of proinflammatory cytokines has on mitochondrial function, it has been demonstrated in various stress paradigms that repeated stress exposure has dramatic effects on mitochondria. For example, chronic immobilization stress and chronic mild stress inhibit the activity of the respiratory chain complexes within the rat brain cortex (Madrigal et al., 2001; Rezin et al., 2008) and was shown to reduce hippocampal Na^+^, K^+^-ATPase activity (Gamaro et al., 2003). Moreover, chronic mild stress has been shown to reduce respiration rates of mitochondria located in the mouse hippocampus, cortex and hypothalamus (Gong et al., 2011). This study also confirmed that stress significantly impacted the ultrastructure of mitochondria (Gong et al., 2011), which are features of mitochondria in presynaptic neurons that have been coupled to changes in synaptic strength (Cserép et al., 2018). Moreover, there is evidence to suggest that distinct differences in mitochondrial function regulate an anxiety-like phenotype. For example, rats that exhibited high anxiety-like behavioral tendencies also demonstrated reduced expression of mitochondrial complex I and II proteins and decreased respiratory capacity and ATP (Hollis et al., 2015). Surprisingly, however, there is a paucity of studies evaluating the impact of social defeat stress on brain mitochondria and even further lack of studies determining whether the vast stress-induced changes in mitochondrial function are driven by stress-induced proinflammatory cytokines.

#### Oxidative Stress/Reactive Oxygen Species (ROS)

Active neurons exhibit high rates of oxygen consumption and as a result, produce large amounts of ROS (Halliwell, 1992). Mitochondria are the energy powerhouse of the cell and represent the largest source of ROS production in addition to monoamine oxidase and nitric oxide synthase. While ROS play a role in several critical neuronal functions such as neuronal plasticity and learning and memory (reviewed in: Massaad and Klann, 2011), the large amounts of ROS are tightly regulated by an antioxidant system. Under conditions where this system becomes unbalanced, a deleterious buildup of ROS is linked to stress-related psychiatric pathology in clinical studies and is demonstrated to occur in stress-related preclinical studies (de Oliveira et al., 2007; Salim et al., 2010, 2011; Lindqvist et al., 2017). Because mitochondria play a critical role in the production and metabolism of ROS, mitochondrial dysfunction is directly related to increased oxidative stress (Mattson et al., 2008). In line with evidence discussed above that TNF-α reduces mitochondrial function, ROS are also dose dependently increased by treatment with either TNF-α or IL-6 (Rochfort et al., 2014). Social defeat stress has also been shown to induce ROS in stress-related brain regions, and moreover ROS have been shown to play a permissive role in the anxiety-like behavior following social defeat in rats (Solanki et al., 2017). Interestingly, rats demonstrating a high anxiety-like phenotype also exhibit increased ROS production within the NAc (Hollis et al., 2015). Finally, lending evidence to the role for ROS in the pathogenesis of psychiatric disorders in humans, depressed patients not only exhibited elevated markers of inflammation and the oxidative stress marker F2-isoprostanes, but compared to individuals who readily respond to antidepressants, non-responders had higher levels of both oxidative stress markers and inflammation (Strawbridge et al., 2015; Vaváková et al., 2015; Lindqvist et al., 2017). Taken together, it is clear that proinflammatory cytokines are capable of shifting the balance of ROS production/elimination from a healthy balance towards maladaptive. However, it is yet to be determined whether stress-induced ROS and subsequent anxiety- and depressive-like behavior is initiated by proinflammatory cytokines and chemokines.

### Glutamate Neurotransmission and Excitotoxicity

The involvement of glutamate has also become an area of interest in the etiology of depression. For example, heightened excitability of hippocampal neurons may underlie the loss of glutamatergic pyramidal neurons in depressed patients (Rajkowska et al., 2005) and evidence from human postmortem tissue has identified alterations in excitatory amino acid transporters (EAATs) 1 and 2 and glutamine synthetase (Rajkowska and Stockmeier, 2013). Moreover, it has been shown that ketamine, a noncompetitive NMDA antagonist (Anis et al., 1983), is capable of producing long lasting antidepressant effects (Berman et al., 2000) even in patients that demonstrate resistance to traditional antidepressant therapies (Zarate et al., 2006). Importantly, the inhibitory action of ketamine requires the presence of open NMDA channels (MacDonald et al., 1987) and can remain bound to NMDA receptors even after the channels have closed (Huettner and Bean, 1988), providing pharmacological validity to these prolonged treatment effects. Several preclinical models have demonstrated that exposure to stress can result in abnormalities in glutamate signaling. For example, 8 weeks of social isolation has been shown to enhance the expression of both NR2A and NR2B subunits within the hippocampus (Chang et al., 2015). Stress-induced increases in these NMDA receptor subunits within the hippocampus are known to not only enhance the intensity of excitatory postsynaptic potentials (Chang et al., 2015) but are also associated with the emergence of aggression, anxiety- and depressive-like behaviors in rodents (Costa-Nunes et al., 2014; Chang et al., 2015). However, it is important to note that these alterations in NMDA receptor subunits following stress exposure are brain region specific. Within the NAc, mice exposed to chronic social defeat that also demonstrate behavioral susceptibility, exhibit long term reductions of NR2B subunit (Jiang et al., 2013). The loss of NR2B subunits significantly impacted the synaptic function of NAc neurons by promoting an increase in long-term depression (Jiang et al., 2013). Interestingly, this study went on to determine that treatment with Fluoxetine, a selective serotonin re-uptake inhibitor, was capable of reversing the effects of defeat stress in susceptible mice such that the molecular profiles within the NAc were nearly identical to mice demonstrating resilience to the effects of social defeat (Jiang et al., 2013).

Stress-induced alterations of NMDA receptors are not the only putative source of glutamatergic excitotoxicity in the brain. For example, unpredictable stress exposure has been documented to produce similar alterations in the subunit composition of α-amino-3-hydroxy-5-methyl-4-isoxazolepropionic acid (AMPA) receptors such that stress exposed rodents demonstrated greater expression of GluR1 subunits (Hubert et al., 2014). Moreover, exposure to the unpredictable stress paradigm resulted in a shift in AMPA receptor distribution such that a greater number of AMPA receptors were found on dendritic spines (Hubert et al., 2014).This seemingly minor shift is well known to produce functionally relevant alterations in neuronal signaling. AMPA receptors which express the GluR2 subunit are impermeable to extracellular Ca^2+^ due to an arginine block. Therefore, loss of GluR2 subunits enhances the signaling strength of AMPA receptors by enhancing the magnitude of the elicited depolarization following AMPA receptor stimulation (Isaac et al., 2007). Taken together, these findings suggest that exposure to unpredictable stress may result in significant remodeling of dendritic spines to vastly increase their sensitivity to excitatory stimuli. These effects, similar to those demonstrated in microglia, require at least 24 h following the cessation of stress to become evident suggesting that these alterations are largely driven by alterations in gene expression (Nasca et al., 2015) and are not associated with the immediate stress response. Interestingly, GluR2 subunits are also known to shift across the lifespan. In rodents it has been shown that GluR2 steadily increases from birth until adulthood. However, this composition of AMPA receptors does not remain stable and does decrease such that 70-week-old rodents exhibit significant decline in both protein and mRNA for the GluR2 subunits within the hippocampus (Pandey et al., 2015). While it is unclear if inflammatory senescence and enhanced inflammatory reactivity is associated with this shift in GluR2 subunits, these data suggest that a natural loss of GluR2 may contribute to the enhanced risk of mood disorders in aging populations.

A number of studies have directly implicated neuroinflammation and microglial processes in the emergence of glutamatergic excitotoxicity (Faust et al., 2010; Diamond and Volpe, 2012). Most directly, glutamate can be released from activated microglia (Barger et al., 2007) or neurons following stimulation with cytokines such as IL-1β in a dose dependent manner (Zhu et al., 2006). In addition, cytokines released by neighboring microglia are capable of acting upon neurons to increase neuronal glutaminase (Ye et al., 2013), a mitochondrial enzyme responsible for the conversion of glutamine to glutamate (Zhao et al., 2012). Importantly, TNF-α-induced increases in glutaminase have been tied to the induction of ROS (Wang K. et al., 2017), demonstrating the functional overlap that exists in these stress and inflammatory sensitive systems. In addition to stimulating the release of glutamate, microglia can actively synthesize and release D-serine (Wu et al., 2004). D-serine is a co-agonist for NMDA receptors and strikingly exhibits a three-fold greater affinity for the receptor compared with glycine (Matsui et al., 1995). Several studies have demonstrated that exposure to social defeat stress in mice is capable of enhancing D-serine that is associated with anxiety- and depressive-like behaviors (Wang J. et al., 2017; Dong et al., 2018). Moreover, genetic deletion of D-serine was capable of conferring resilience to mice exposed to chronic social defeat (Dong et al., 2018). While it is currently unclear if these defeat-induced increases in D-serine are driven by defeat-induced proinflammatory cytokines or activation of microglia, it has been shown that administration of nonsteroidal anti-inflammatories such as mefenamic acid (Armagan et al., 2012a,b), acetaminophen, and naproxen (Armagan et al., 2012a) are capable of inhibiting D-serine.

Importantly, the role of stress and inflammation in glutamatergic excitotoxicity extends beyond glutamate receptors and their ligands. A number of studies have further demonstrated microglial involvement in glutamate accumulation in the extracellular space. Specifically, microglial stimulation with IL-1β (Ye et al., 2013) or TNF-α (Takeuchi et al., 2006; Ye et al., 2013) promotes the release of microglial glutamate. Under normal resting non-stress conditions, the brain has a number of mechanisms in place to manage excess synaptic glutamate. One such method is astrocyte mediated uptake via EAAT1 and EAAT2 in humans and glutamate–aspartate transporter (GLAST) and glutamate transporter 1 (GLT1) in rodents (Bezzi et al., 2004; Furuta et al., 2005). However, this protective mechanism has been shown to fail in instances where glutamate accumulation resulted from stimulation of microglia. Specifically, accumulation of glutamate in astrocytes results in a compensatory downregulation of EAAT1 (Takaki et al., 2012). Although preclinical studies assessing the role of GLAST and GLT1 in social stress-induced behavioral dysfunction has not been assessed, clinical studies have demonstrated altered expression of EAAT1 and 2 within brains of depressed patients (Miguel-Hidalgo et al., 2010; Rajkowska and Stockmeier, 2013). Together these data indicate that cytokine activation of microglia may result in a complex dysregulation of glutamate neuronal transmission by both enhancing local glutamate synthesis, stimulating glutamate release, and indirectly resulting in a downregulation of receptors involved in the maintenance of extra synaptic glutamate.

### Remodeling of Excitatory Synaptic Terminals

In addition to modifying the release, synthesis and uptake of glutamate, stress and inflammation are known to alter the structure of excitatory synaptic terminals. Specifically, it has been shown that chronic stress results in the loss of dendritic spines in areas such as the prefrontal cortex (Goldwater et al., 2009). This loss of spines is directly associated with the emergence of anxiety- and depressive-like behaviors (Qiao et al., 2016). Stress has further been postulated to contribute to these effects by modulating a number of factors including the synthesis and release of MMP-9. In addition to promoting disruptions in the BBB (see “Blood Brain Barrier Disruption” section), MMP-9 is also involved in synaptic plasticity and remodeling of dendritic spines (Wang et al., 2008). In the presence of MMP-9, dendritic spines reshape from a short and round to a long and thin morphology (Michaluk et al., 2011). These long and thin spines are suggested to be less effective in conducting excitatory signals as they restrict Ca^2+^ flow (Ebrahimi and Okabe, 2014). Moreover, the thin and elongated spines also demonstrate greater vulnerability to the damaging cellular consequences of stress exposure (Radley et al., 2008; Bloss et al., 2011). In this manner, MMP-9-induced remodeling of dendritic spines may reduce neuronal excitability and promote the loss of dendritic spines. While clinical studies documenting the role of MMP-9 in the emergence of stress-induced depression are lacking, preclinical studies have demonstrated that exposure to social defeat results in elevations of the cytokine IL-1α and MMP-9. Importantly, these findings were most pronounced in susceptible rodents (Stelzhammer et al., 2015). These effects of social defeat on dendritic spines is not limited to MMP-9. Within the NAc inhibition of κB kinase (IκK) has also been shown to promote the formation of long and thin spines in animals exposed to social defeat (Christoffel et al., 2012). Moreover, this study found a trend to suggest that a greater number of long and thin spines was negatively associated with social interaction which could be reversed by inhibiting IκK (Christoffel et al., 2012). This same group later showed that chronic exposure to social defeat was also associated with an increase in the number of immature stubby spines in the NAc (Christoffel et al., 2015). In accordance with their previous findings, a larger number of stubby spines was associated with the emergence of social avoidance (Christoffel et al., 2015).

In the developing brain, microglia are well known to contribute to the remodeling of neuronal synapses through a process termed synaptic pruning (reviewed in Lenz and Nelson, 2018). Synaptic pruning has been described as a phagocytic event where immature and highly active synapses are permanently removed. It was originally suggested that this occurred via microglial engulfment of dendritic spines. However, a study by Weinhard et al. (2018) demonstrated that although microglia did contact dendritic spines, they did not completely engulf the dendritic spines for elimination. Instead it was found that microglia participate in a process termed trogocytosis in which only a small portion of the dendritic spine is phagocytosed (Weinhard et al., 2018). This process of trogocytosis also stimulates the formation of new long and thin filopodia shaped spines (Weinhard et al., 2018). While studies determining the involvement of microglial pruning in the consequences of stress exposure is unknown, it is probable that similar phagocytic processes could occur as a consequence of stress exposure.

## Conclusion

Prospective studies have clearly linked inflammatory related disorders with increased risk of depression. Moreover, several clinical studies support the notion that neuroinflammation is associated with depressive symptomatology. However, our understanding of the mechanisms that are impacted by neuroinflammation, especially in the context of social stressors is at its infancy. Gaining a better understanding of neuroinflammatory-mediated adaptations that occur during stress and are capable of producing psychopathology will be a great advance in understanding the role of neuroinflammation in the etiology of depressive and anxiety disorders. Beyond the recognized effects of inflammatory cytokines on neurotransmitter and neuropeptide systems, inflammation may likely regulate susceptibility to social stress by altering the BBB, sensitizing microglia, producing mitochondrial dysfunction and oxidative stress as well as contributing to glutamate toxicity. This review highlights these cytokine-sensitive mechanisms that are favorably positioned to contribute to pathology, yet in many cases their direct regulation by inflammatory cytokines in the context of social stress has not been determined and will represent a great advance to the etiology of stress-induced psychiatric disorders.

## Author Contributions

SW researched and wrote a considerable amount of the review article (2.5–3 of the 5 sections) and edited the rest of the review written by JF. JF researched and wrote a large amount of this review article (2.5 of the 5 sections), and revised the document as suggested.

## Conflict of Interest Statement

The authors declare that the research was conducted in the absence of any commercial or financial relationships that could be construed as a potential conflict of interest.
